# The role of the common agricultural policy in contributing to jobs and growth in EU’s rural areas and the impact of employment on shaping rural development: Evidence from the Baltic States

**DOI:** 10.1371/journal.pone.0262673

**Published:** 2022-02-03

**Authors:** Tomasz Grodzicki, Mateusz Jankiewicz

**Affiliations:** Nicolaus Copernicus University in Torun, Faculty of Economic Sciences and Management, Torun, Poland; Murdoch University, AUSTRALIA

## Abstract

Agriculture is a very important sector of the economy that has a great influence on rural areas. Almost sixty years of the Common Agricultural Policy (CAP) in the European Union (EU) may impose a question on the impact of this policy on job creation and economic growth in rural areas. By analyzing key indicators, one should note that although there are still many problems to tackle, the CAP has been successful to some extend in increasing employment rate and Gross Domestic Product (GDP) per capita as well as decreasing poverty rate in the EU rural areas. There are also more and more households connected to the next-generation broadband network and there is still a significant number of bed places in the EU rural areas. Although, the situation in specific EU Member States and their rural regions may be different, the overall performance of the EU is satisfactory and shows positive prognostics for the current EU financial perspective of 2021–2027. This paper also provides an empirical approach which checks whether there is a connection between the employment rate and the rural development measured in GDP per capita based on the data from the Baltic States: Estonia, Latvia and Lithuania in the period of 2000–2020. This research confirms that when increasing the level of rural employment, the economic growth rises in rural areas.

## 1. Introduction

Agricultural has always been one of the European Union’s main policies. It is managed by the so-called the Common Agricultural Policy (CAP). The CAP consists of two ‘pillars’. The first one is designed for income subsidies and other market interventions. It includes direct payments (in order to help farmers stabilize farm revenues which are put at risk annually due to volatile market prices and different weather conditions) and market measures (in order to address specific market externalities and to promote trade). The second pillar has been designed for rural development policy. It concerns various activities such as: encouraging rural initiatives, supporting young farmers, helping farmers with both diversification and setting up producer groups, or alternatively restructuring their businesses.

Agricultural sector does affect the following aspects: society, environment and economy. Therefore, it is important to focus on these three paths that together can contribute to one common goal leading to sustainable agriculture. These all three pillars are connected in such a way that farmers earn a living when producing goods which are made using natural resources. Once they sell agricultural products, they earn money so they can make a living and they feed the society. This process of agricultural activity sustains not only farm families and rural communities but also society as a whole.

Changes in the natural environment (changing weather conditions, droughts or floods) are more and more noticeable in the EU. Farmers have to deal with their consequences, i.e. with the economic effects they bring, i.e. lower yields and, consequently, a decrease in income from agricultural production. The task of the CAP is therefore to counteract the negative economic effects caused mainly by changes in the natural environment. For this purpose, the following measures have been introduced to protect the profitability of agriculture and at the same time protect the environment [1]:

- implement a fair income support system and achieve greater equity in the agri-food supply chain,
- cross-compliance and greening payments, which will strengthen the links between income support and environmental measures
- providing support for the development of rural areas, including economically sustainable environmental practices and investments for farmers.

The main task of CAP after 2020 is to ensure constant access to high quality food as well as to support the European model of agriculture, which will be outlined in the following nine key objectives [2] (p.3):

1. ‘Support viable farm income and resilience across the Union to enhance food security.
2. Enhance market orientation and increase competitiveness.
3. Improve the farmers’ position in the value chain.
4. Contribute to climate change mitigation and adaptation, as well as sustainable energy.
5. Foster sustainable development and efficient management of natural resources such as water, soil and air.
6. Contribution to the protection of biodiversity, enhance ecosystem services and preserve habitats and landscapes.
7. Attract young farmers and facilitate business development in rural areas.
8. Promote employment, growth, social inclusion and local development in rural areas, including bio-economy and sustainable forestry.
9. Improve the response of EU agriculture to societal demands on food and health, including safe, nutritious and sustainable food, as well as animal welfare.’

The viability of rural regions is to a large extent provided by the employment opportunities [3, 4]. Thus, this paper focuses on specific objective number 8 which is to contribute to jobs and growth in the EU rural areas. The key messages of this objectives are as follows [5]:

- Predominantly rural areas covered approximately 44% of the EU and their population accounted for 19% in 2018 (including Great Britain at that time). Most of these regions are characterized by significantly lower level of income per capita than the average in the EU, since they mainly rely on the primary sector due to the close links in terms of employment.
- The level of poverty as well as the share of poor people in the population was higher in rural regions but it is difficult to generalize at the EU level since there are significant differences between particular EU Member States.
- Rural areas that stay in isolation (far from urban regions) may suffer from social exclusion and limited opportunities in a labor market. Moreover, access to the broadband infrastructure is crucial for economic activities prospects in rural areas, especially concerning employment and entrepreneurship.
- The CAP is designed to tackle unemployment and poverty in rural areas, in particular through the positive effects of decoupling and rural development measures.

Hence, it is vital to assess the CAP in the context of its contribution to jobs and growth in rural areas as well as to look at the specific case (of the Baltic States) and the relation of employment with rural development.

## 2. Materials and methods

The first part of the analysis concerns the EU’s rural areas and the role of the CAP. Since, there are nine specific objectives to measure performance of the Common Agricultural Policy, one of them is jobs and growth in rural areas. This paper considers the following data used for the research: employment rate, GDP per capita, households with the next generation access (NGA) broadband coverage, poverty rate, and the total number of bed places in tourism. This research uses the combination of both: the literature review with discussion on some specific issues and data taken from publicly available sources that are incorporated in the form of graphs and figures. Such an approach can clearly lead to formulate the current state of knowledge on the economic growth in rural areas, and then to point out for the future directions and improvements.

In the second part of this paper, there is an empirical study, which focuses on the study of employment and GDP per capita in the Baltic States: Estonia, Latvia, Lithuania. In the analysis of each process given in the form of a time series, we can use their transformation into the frequency domain. The advantage of this approach is the ability to quickly identify dominant periods (or frequencies) in the time series. Therefore, in the first part of the analysis, the evaluation of the periodogram characteristics for every of the considered processes is done. Based on these characteristics, the presence of the non-stationarity of the average features is evaluated. This type of non-stationarity is concerned with the occurrence of a deterministic trend.

In order to filter out the processes from the long-term tendencies, which conform to non-stationarity of the average, the classical trend models are used. These models are estimated in the time domain and take the following form:

$$y_{t}=\alpha_{0}+\alpha_{1}t+\varepsilon_{t},$$ (1)

$$y_{t}=\alpha_{0}+\alpha_{1}t+\alpha_{2}t^{2}+\varepsilon_{t},$$ (2)

where *y*_*t*_ denotes the implementation of the process *Y*_*t*_ and *t* and *t*^2^ are linear and squared time variable respectively. Moreover, *α*_0_, *α*_1_, *α*_2_ are the structural parameters of the models, but *ε*_*t*_ is the random component.

After filtering out the processes from non-stationarity of the average, the presence of trend in the variance of processes is investigated. If it occurs, processes exhibit the features of the non-stationarity of the variance (processes are difference-stationary). Thus, in order to test the occurrence of the unit root, the augmented Dickey-Fuller test (ADF) is used. Test is based on the following auxiliary model:

$${\Delta y}_{t}=\delta y_{t-1}+\sum_{i=1}^{p}\gamma_{i}{\Delta y}_{t-i}+\varepsilon_{t},$$ (3)

where Δ operator denotes first differences of the time series (Δ*y*_*t*_ = *y*_*t*_−*y*_*t*−1_). The null hypothesis of the test is formulated as *H*_0_: *δ* = 0 and it proclaims that in the formation of the process the unit root occurs. To confirm the conclusions from the ADF test, the stationarity of the processes with the KPSS test is additionally checked.

In the next step, after reducing processes *Y*_*t*_ and *X*_*t*_ to the stationarity, the relationship model between the considered processes is estimated, gived as:

$$Y_{t}^{s}=\beta_{0}+\beta_{1}X_{t}^{s}+\sum_{j=1}^{q}\gamma_{j}Y_{t-j}^{s}+\varepsilon_{t}.$$ (4)

In the model (4) $Y_{t}^{s}$ and $X_{t}^{s}$ are processes which are reduced to the stationarity form of *Y*_*t*_ and *X*_*t*_. Part $\sum_{j=1}^{q}\gamma_{j}Y_{t-j}^{s}$ is inserted to exclude the autocorrelation from the model residuals. The quality of the model is evaluated with the use of the following diagnostic tests: (1) White test, verifying the residuals homoscedasticity, (2) Jarque’a-Bera test (JB), verifying the normality of the residuals distribution and (3) CUSUM test, checking the time stability of the model parameters.

Data used in the analysis concern the Gross Domestic Product of the rural areas per capita (*Y*_*t*_) and the employment rate at the rural areas (*X*_*t*_) in the period of 2000–2020. All calculations are made in the Gretl software, but figures are prepared with the use of the Python software.

In the both parts of this study, the data are applied from the Eurostat.

## 3. Results and discussion

Many studies have signalized the problem of inequality in direct payments distribution [6–9]. According to official data around 20% of all beneficiaries get approximately 80% of the total direct payments. The problem with direct payments is that they are largely area-based support so their distribution is designed by the concentration of land– 20% of the biggest agricultural holdings in the EU concentrate 82% of overall agricultural land [10].

**Fig 1:**
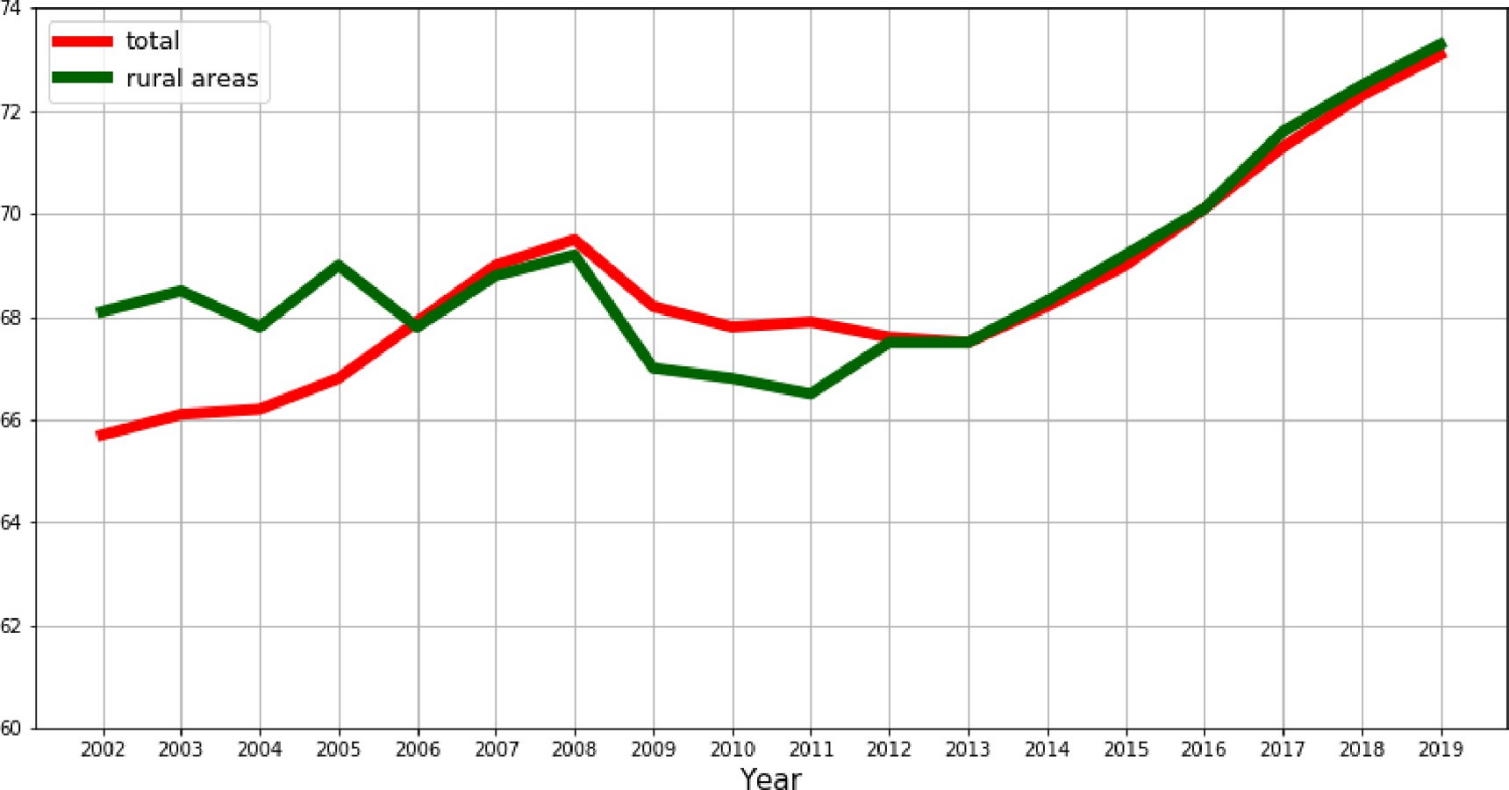
Employment rate for the age group 20–64 in the European Union (EU).

Fig 1 shows the level of employment rate for the age group 20–64 in the EU. In the period of 2002–2005, the employment rate for the age group 20–64 was higher in rural areas than in the overall EU economy, while in the period of 2006–2012, there was an opposite relation. During and a few years after the financial crisis, the employment rate decreased both in rural areas and in general in the EU. The employment rate in the EU rural areas steadily increased from 67.5% in 2013 to 73.1% in 2019. The employment rate in the EU steadily increased from 66.5% in 2011 to 73.3% in 2019 while the employment rate in total EU economy followed a similar upward trend. Interestingly, in 2015 the EU managed to get back to the employment level before the 2009 financial crisis.

The level of development of rural areas, to a large extent, determines the state of the EU economy, and at the same time impacts the quality of life of its inhabitants. However, forest areas contribute to overcoming the negative consequences of climate change. Therefore, it is difficult to find a right balance on what to do in terms of rural areas. The problem arises on whether the policy should focus on developing rural areas or to leave them as they are and prevent deforestation practices. In many rural areas in the EU countries there are social and economic problems that are more severe than in urban areas, such as: limited access to certain services and infrastructure, poverty that results in the outflow of young people and the aging problem of the society [11–13].

In the EU, the situation of the labor market in rural areas is very diverse, with the noticeable disparity between Western and Central and Eastern European economies previously undergoing transformation processes. This is mainly due to: (1) demographic factors shaping the structure of the labor supply in rural areas, (2) economic conditionings like labor productivity in agriculture or tempting employment opportunities outside agriculture, (3) regional characteristics including tourist attractiveness and access to specific services and resources [14].

Before analyzing the GDP per capita in rural areas in the EU, one should note that one of the CAP’s aims is to diminish the gap between rural and other areas in standard of living in the EU. Rural areas are vital for expanding economies activities there since focusing only on urban areas is not sustainable in the long run. Too much concentration of economic activity in cities may lead to natural resources exploitation what then may worsen the quality of life in urban areas. Therefore, attracting young people to stay and work in rural areas is of great importance for achieving economic convergence in the regions of the EU. This is also the aim of the EU’s Cohesion policy which cannot be overestimated in the process of reducing the gap between less developed and more developed regions, what is very often referred as territorial cohesion [15–17]. The combination of the Cohesion policy and the CAP plays an important role in the regional development of rural areas [18–21].

**Fig 2:**
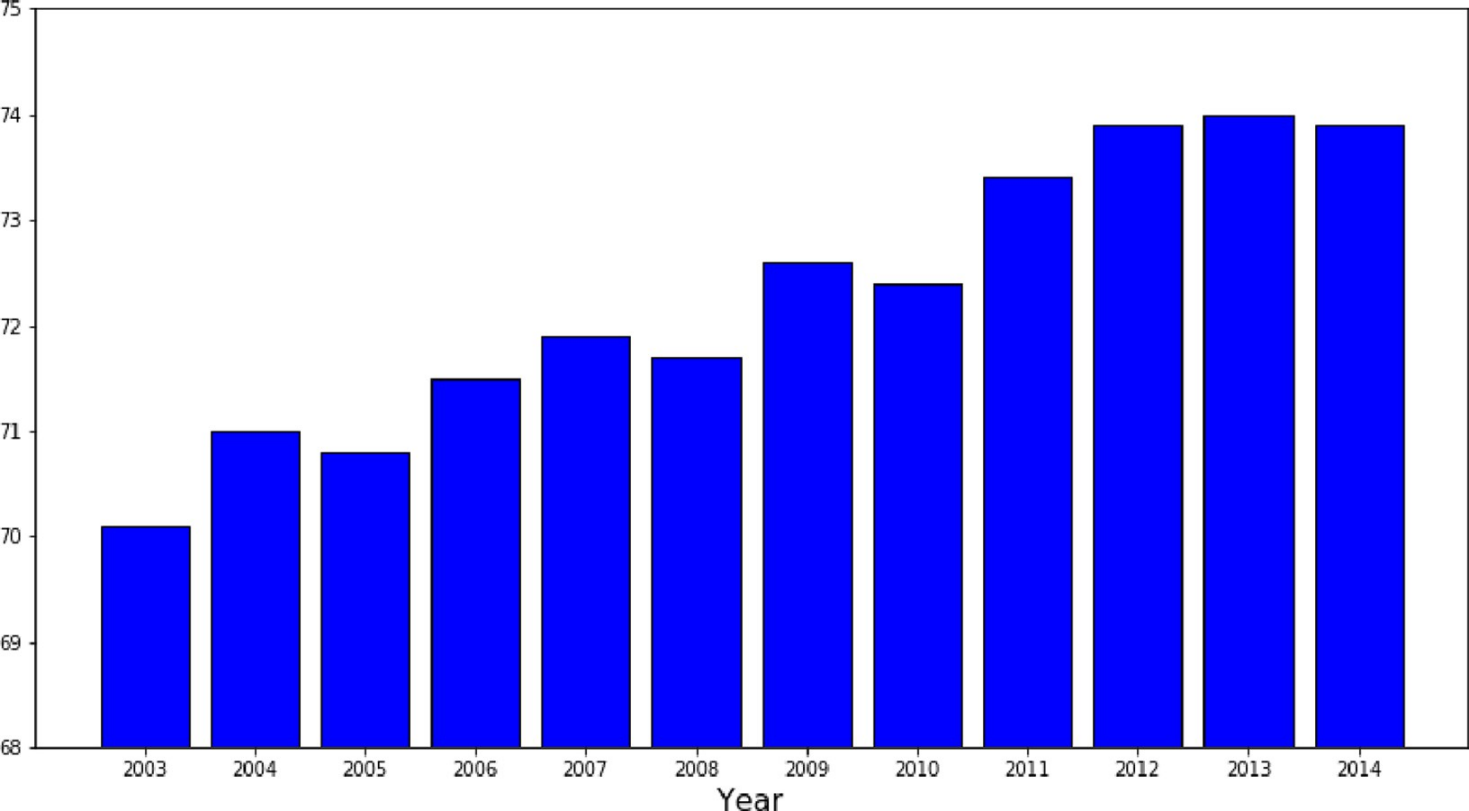
Gross Domestic Product (GDP) per capita in rural areas indexed to total GDP (in PPS) in the EU (EU = 100).

Therefore, GDP per capita in purchasing power standard (PPS) may be applied to compare the aggregate standard of living in different territorial dimensions. Fig 2 shows the total GDP per capita in rural areas indexed to total GDP in the EU. The results indicate that the level of GDP per capita in rural areas in the EU was 26–30 index points below the total EU’s GDP per capita in the period of 2003–2014. This upward trend implies that there might be a process of convergence between rural regions and overall EU economy.

**Fig 3:**
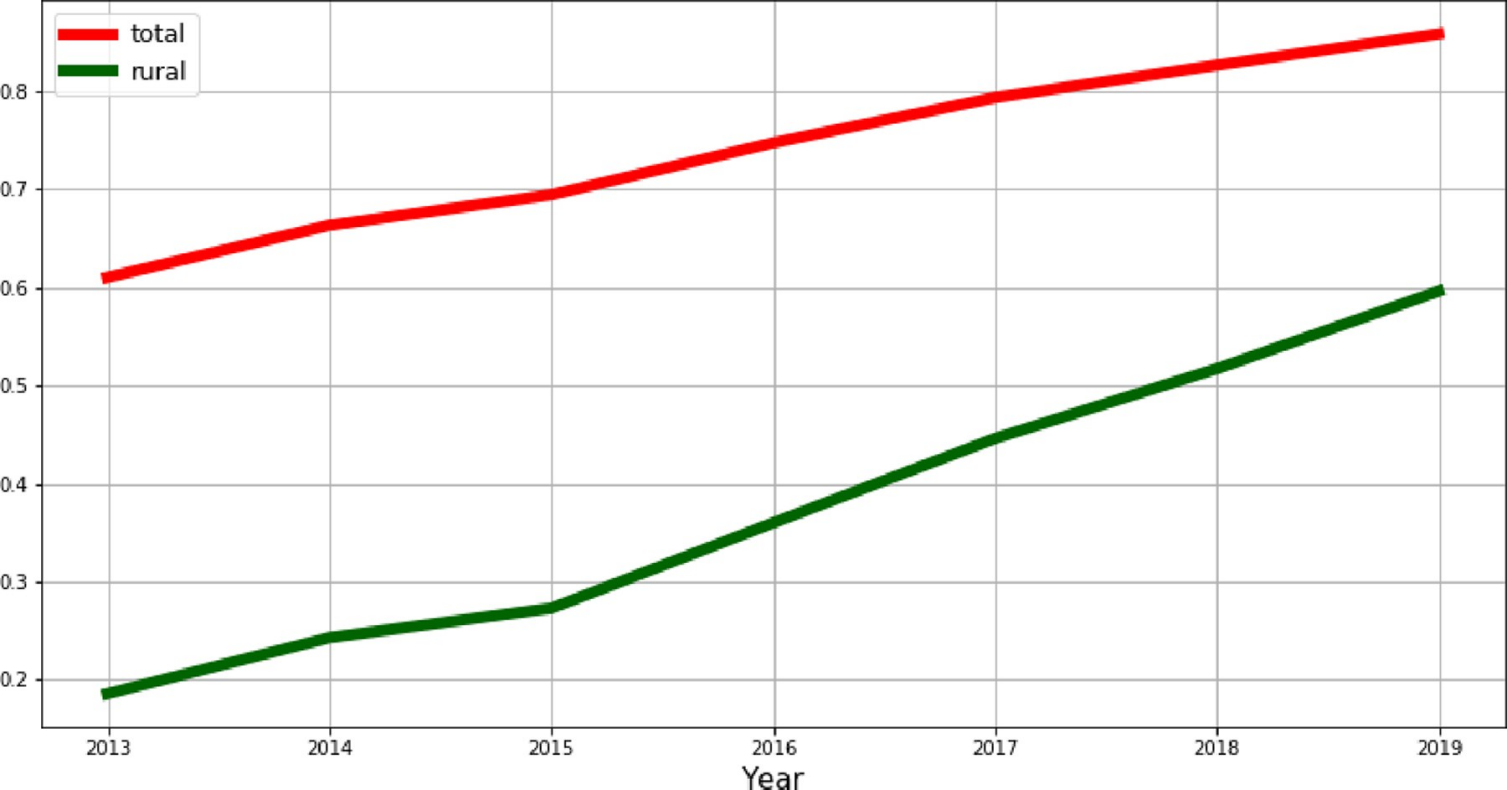
Households with an access to the next-generation (NGA) broadband coverage in EU rural areas.

Fig 3 shows the share of households with an access to the next generation (NGA) broadband. There has always been a gap between rural and urban areas in an access to broad-band network. In 2019, 59.6% of rural households were equipped with a Next Generation Access (NGA) network, in comparison to 85.8% of total EU households. Albeit, there was a significant improvement in NGA in rural areas over time (from below 20% in 2013 up until nearly 60% in 2019), but reducing the rural-urban connectivity gap is still treated as a challenge for policymakers.

**Fig 4:**
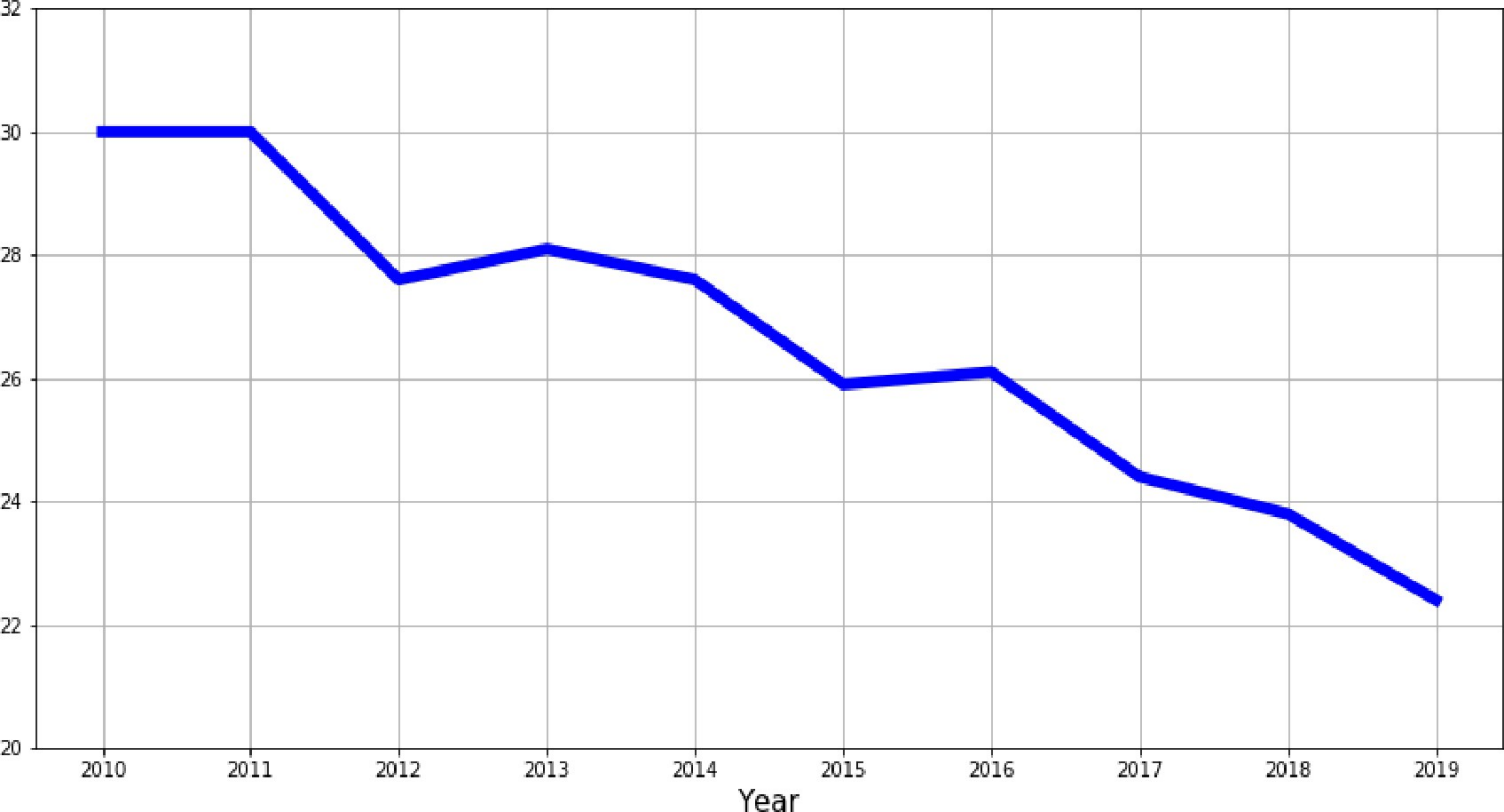
Poverty rate in rural areas in the EU (% of total population).

Poverty is also an important aspect that, in turn, negatively affects economic growth in rural areas. Its rate is defined as the share of population at risk of poverty or social exclusion. Fig 4 shows that the rural poverty rate in the EU declined from 30% in 2010 to 22.4% in 2019. Although this downward trend a positive sign for the EU rural areas, one should mind that rural poverty remains a challenge in many places in the EU. Particularly, there are two reasons why this optimistic results from declining trend of rural poverty should be treated with caution. First, there is an ongoing phenomenon of migration to suburban areas. This might take a form of simple intermediate zones between rural and urban areas [22, 23] or even more developed concepted of peri-urbanization [24–27]. Second, the rural poverty very much differs in the EU, and in some countries even more than a half of poor people live in rural areas, e.g. in Croatia, Hungary, Lithuania, Poland, Romania [28–30].

**Fig 5:**
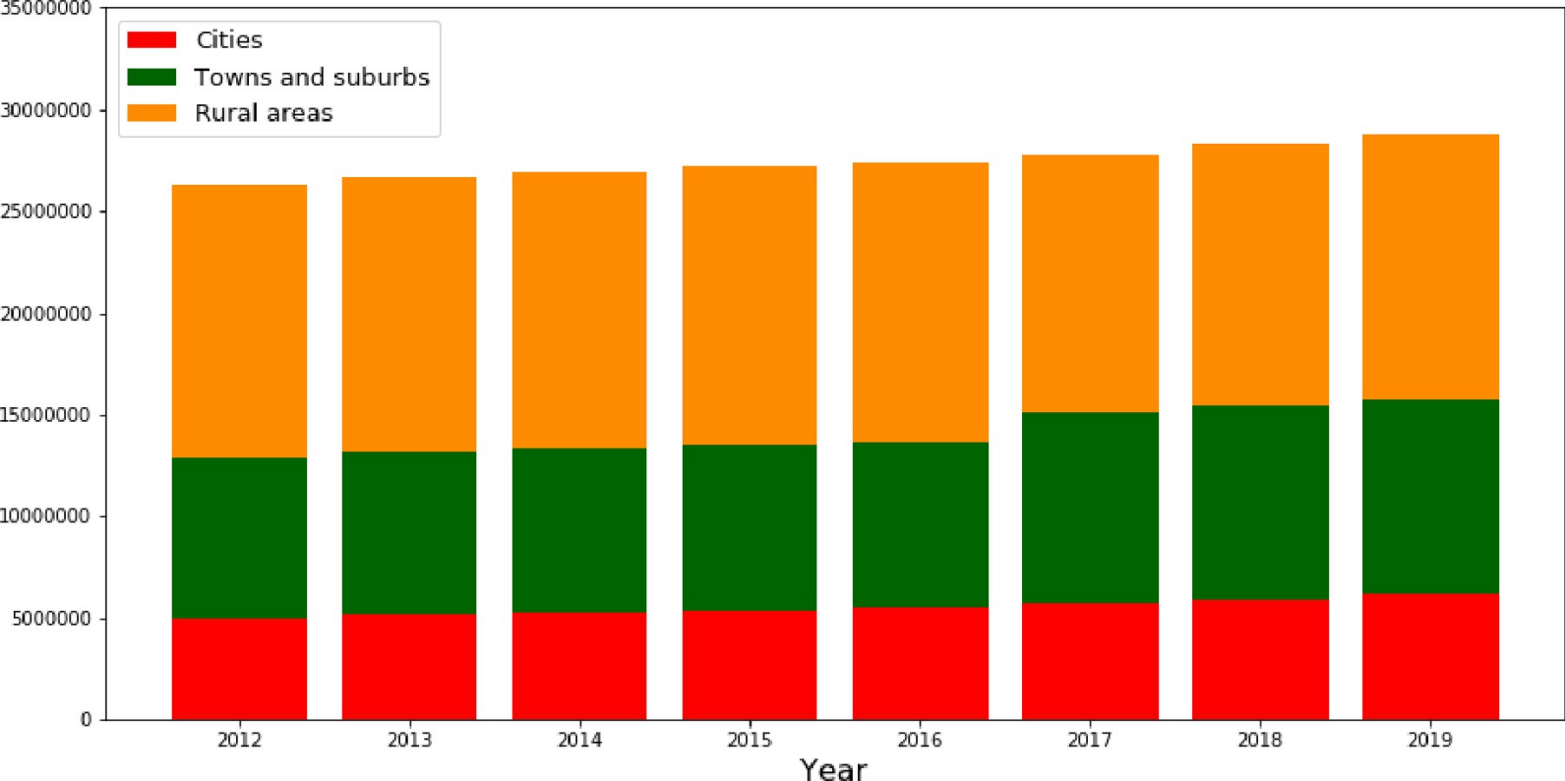
Total number of bed places by type of regions in the EU.

Tourist trips to the countryside are becoming more and more popular and relate to society as a whole. There are many reasons for that including: ongoing urbanization process, more free time, higher wages, better and wider access to education, better transportation, cleaner air, etc. Rural areas provide more and more diversified forms of tourism from a small-scale to the large-scale economic activities in rural tourism. Undoubtedly, increasing importance of tourism in the countryside has boosted the economic growth in rural areas and contributed to jobs creation in rural communities [31–33]. Fig 5 shows that in rural areas there were around 45% to 51% of total bed places available in truism sector in the period of 2012–2019. Although, the total number of bed places slightly decreased in rural areas, they still remain as a crucial tourism destination.

There are also numerous initiatives that help to achieve this objective with regard to contributing to growth and jobs in rural areas. In different EU financial perspectives, there were different names for programs for rural development such as: LEADER I, LEADER II, LEADER +, Rural Development Program (RDP). These programs have aimed at creation of new economic activities, revitalization of living standard, transfer of know-how and innovation, preservation of regional identity and culture of rural areas, etc. One of the very specific initiatives are revitalization programs designed for the purpose of: (1) spatial sphere which include reconstructing the technical infrastructure, and adjusting the spatial organization of the region to its surroundings, and also (2) social sphere in order create a mental environment of revitalization which is of great importance for building a ground for deep and long-term changes in regional economy. Thus, social revitalization may be affected in many ways, i.e. by changing the social structure (professional, educational and demographic), reducing the poverty rate, social exclusion, and preserving the cultural heritage [34–37].

The second part of this research concerns the employment rate and GDP per capita in the Baltic States in the period of 2000–2020.

**Fig 6:**
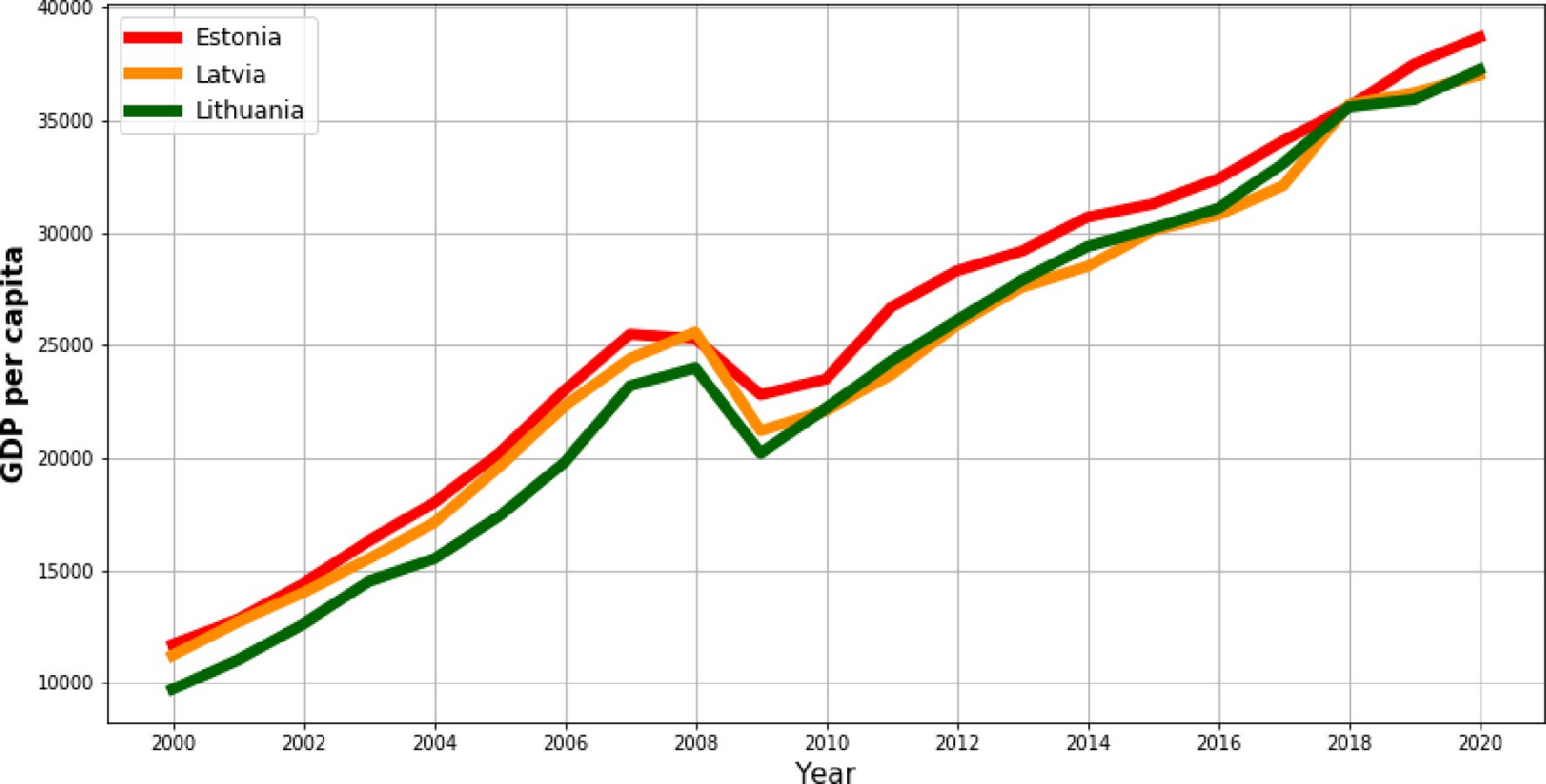
The tendency of the GDP per capita in the Baltic countries in the years 2000–2020.

Fig 6 shows the formation of the GDP per capita values in the whole given period of the research. In each of three Baltic countries the analogous tendency is observed. Until 2008, the level of the considered process was increasing. The only one year of the decline in the GDP per capita was 2009, which to a large extent was caused by the global financial crises, after which the process returned to the upward trend.

**Fig 7:**
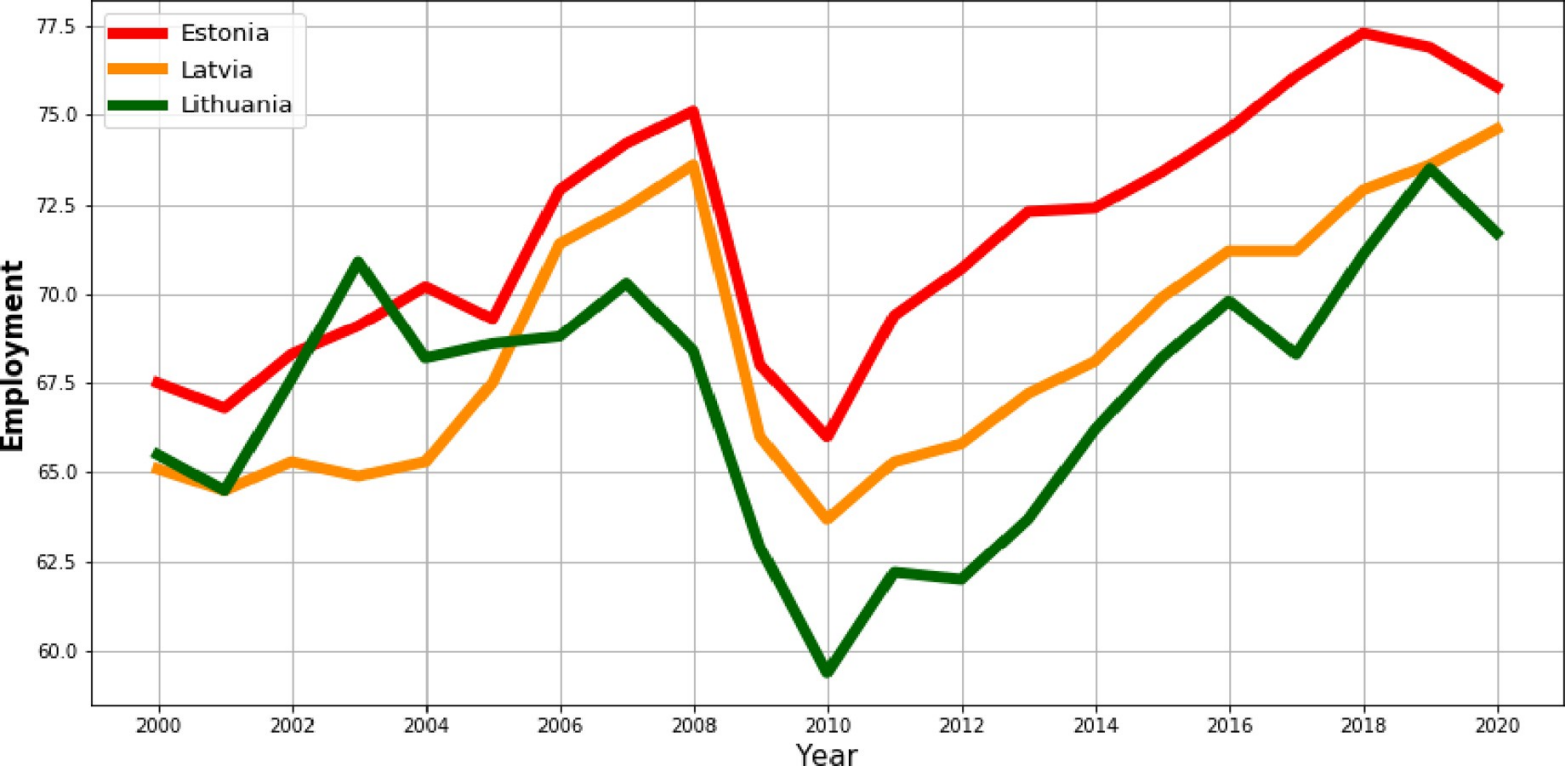
The tendency of the employment rate at rural areas in the Baltic countries in the years 2000–2020.

In turn, Fig 7 shows the evolution of the employment rate in rural areas. Here, by analogy with the previous process, in 2009 there was a slide drop in the employment rate. Nevertheless, the downward trend continued in the following year. The largest fluctuations in the value of the employment rate can be observed in Lithuania, where employment in rural areas is the lowest for most of the analyzed period. For the other two countries, the entire period under consideration can be divided into three phases: the increase in value in 2000–2008, the decrease in value in 2009–2010 and the re-increase in the last 10 years of the analysis.

Based on the presented tendencies in the distribution of the values of processes in time, we can assume that they are non-stationary. Hence, it is difficult to assess the nature of non-stationarity.

**Fig 8:**
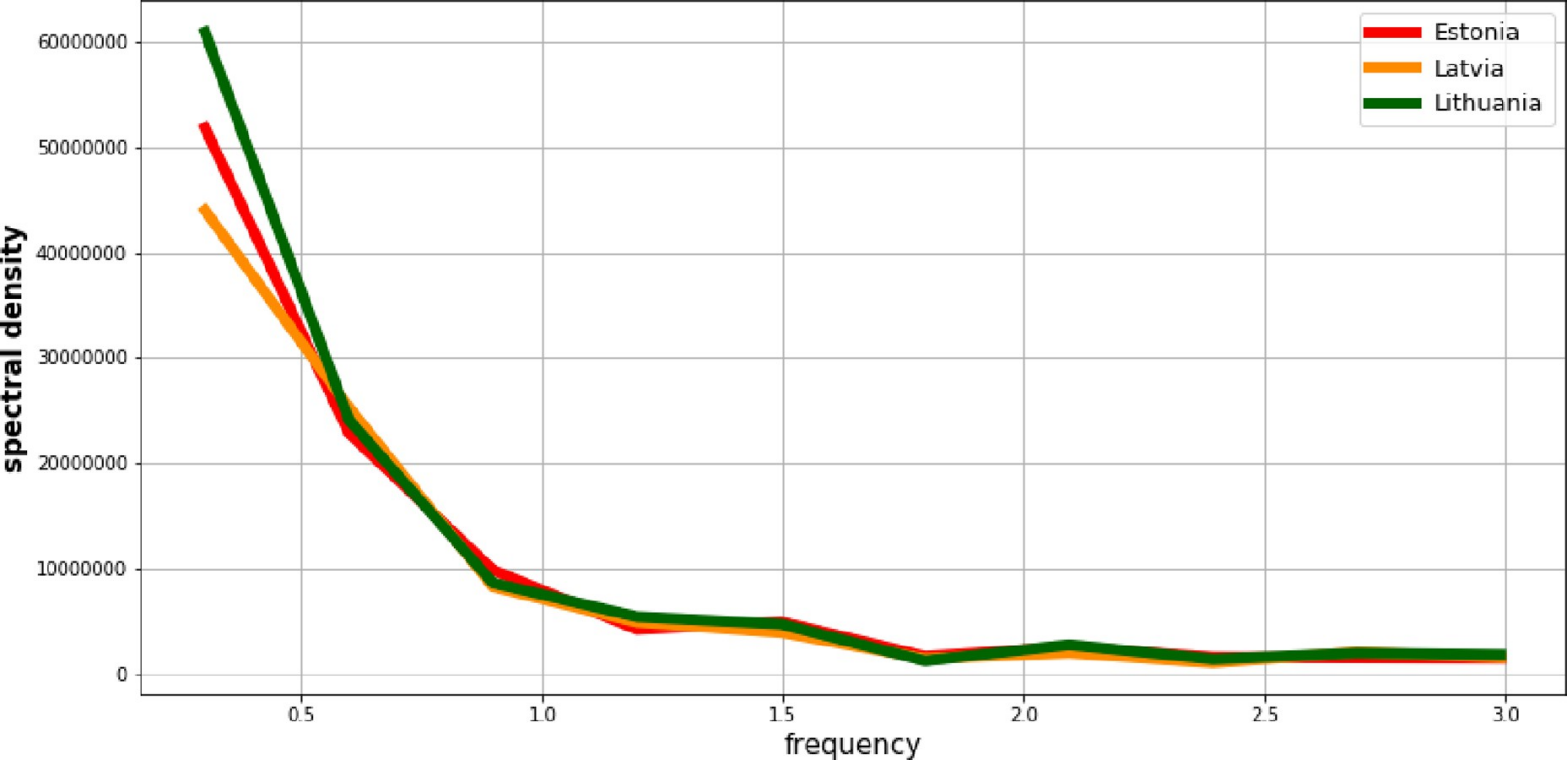
Power spectral density of the GDP per capita process.

**Fig 9:**
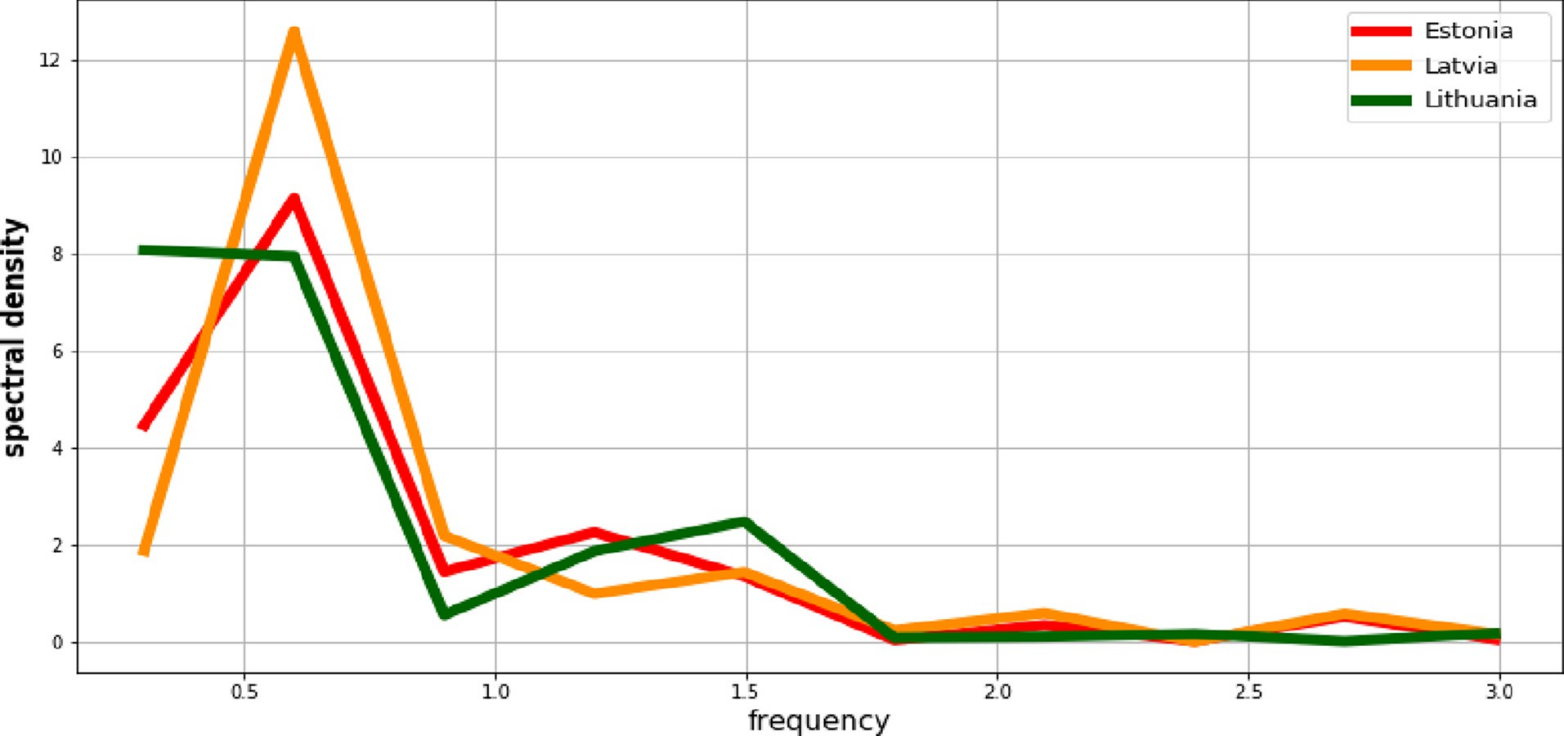
Power spectral density of the employment rate process.

Next, the values of the observation intensity in the frequency domain based on the periodograms are evaluated. Figs 8 and 9 present power spectral density for the processes *Y*_*t*_ and *X*_*t*_ respectively. Each picture shows the situation in all three Baltic States.

Based on the graphs from Figs 8 and 9, the highest share of observations with low frequencies should be indicated. This situation corresponds to presence of the certain long-term tendencies. So both of the processes exhibit features of the trend-stationary processes (non-stationary of the average). Therefore, in order to reduce them to stationarity, models of the deterministic trend are estimated. Table 1 presents the results of their estimation and verification.

**Table 1:** The results of estimation and verification of the trend models for the processes *Y*_*t*_ and *X*_*t*_.

Country	Estonia		Latvia		Lithuania	
**GDP per capita (Y)**						
**Parameter**	**Estimate**	**p-value**	**Estimate**	**p-value**	**Estimate**	**p-value**
** *α***_**0**_	11614.7000	0.0000	11129.0000	0.0000	9105.3200	0.0000
** *α***_**1**_	1273.1900	0.0000	1210.6900	0.0000	1341.0400	0.0000
** *R*** ^**2**^	0.9698		0.9514		0.9763	
**Employment rate (X)**						
** *α***_**0**_	67.0614	0.0000	64.4890	0.0000	70.1866	0.0000
** *α***_**1**_	0.4243	0.0001	0.3690	0.0020	-1.0550	0.0427
** *α***_**2**_	-	-	-	-	0.0548	0.0194
** *R*** ^**2**^	0.5626		0.4041		0.3162	

In most of the cases, the increasing linear trend characterizes the tendency of the processes values changes. Parameter α_1 is statistically significant and its estimate is positive. It denotes the average increase of the GDP per capita values and employment rate in the rural areas in the whole considered period. Quadratic trend is noted only for the employment rate in Lithuania, where the fluctuations of the values were the largest.

Moreover, for the processes filtered out from the deterministic trend, the occurrence of the stochastic trend is investigated. Table 2 contains the results of the ADF and KPSS tests, examining the presence of the unit root and stationarity respectively. The results of the ADF test denote lack of the unit root in all considered processes. The analysis using KPSS test confirms the belief that in the processes filtered out from deterministic trend, the stochastic trend does not occur, so it means that they are stationary.

**Table 2:** The results of the ADF and KPSS tests for the processes *Y*_*t*_ and *X*_*t*_.

Country	Estonia		Latvia		Lithuania	
**GDP per capita (Y)**						
**Test**	**Statistic**	**p-value**	**Statistic**	**p-value**	**Statistic**	**p-value**
**ADF**	-3.1811	0.0014	-3.7998	0.0001	-4.9697	0.0000
**KPSS**	0.0854	> 0.1000	0.0796	> 0.1000	0.0695	> 0.1000
**Employment rate (X)**						
**Test**	**Statistic**	**p-value**	**Statistic**	**p-value**	**Statistic**	**p-value**
**Statistic**	-3.1142	0.0018	-2.6544	0.0077	-2.9703	0.0029
**p-value**	0.0837	> 0.1000	0.0862	> 0.1000	0.0860	> 0.1000

Finally, the models of the relationship between the economic growth and employment rate at the rural areas, defined by (4), for each country are estimated. Table 3 presents the results of estimation and verification of these models. Parameter *β*_1_ in the whole models is statistically significant. Its positive value denotes the average increase in the process $Y_{t}^{s}$ values caused by the increase in the process $X_{t}^{s}$ values. Therefore, an improvement in the employment rate at the rural areas causes the economic growth at these areas. The diagnostic test show desired properties of the models. The exception constitutes lack of the homoscedasticity in the model for Lithuania.

**Table 3:** The results of the estimation and verification of the relationship model between processes $Y_{t}^{s}\;and\;X_{t}^{s}$.

Country	Estonia		Latvia		Lithuania	
**Parameter**	**Estimate**	**p-value**	**Estimate**	**p-value**	**Estimate**	**p-value**
***β***_**1**_	349.0010	0.0015	555.5200	0.0000	299.8710	0.0003
***γ***_**1**_	0.3319	0.0466	-	-	-	-
**Diagnostics**						
**Test**	**Statistic**	**p-value**	**Statistic**	**p-value**	**Statistic**	**p-value**
**White**	2.1264	0.8314	1.4520	0.4838	6.7119	0.0349
**JB**	1.5277	0.4659	1.4835	0.4763	0.2697	0.8738
**CUSUM**	0.2610	0.7972	0.0831	0.9347	0.5996	0.5558

## 4. Conclusions

Promoting employment, growth, social inclusion and local development in rural areas, including bioeconomy and sustainable forestry is one of the ninth key objectives of the CAP after 2020. The CAP will celebrate its 60th anniversary in next year and there are new challenges ahead. The EU rural areas need to be treated with special tools since there are numerous changes caused by i.e. geopolitical situation, natural environment–climate change, social and economic problems, etc.

Over analyzed periods, the EU rural regions experienced decreasing poverty rate; increasing: employment rate and GDP per capita, number of households connected to the next generation broadband network; and a quite stable number of bed places in rural tourism.

Although, analyzing only rural areas at the EU level has some limitations (the results show only the EU average, the situation may be very much different in selected rural regions), the overall picture shows positive future developments of the economic activities in the EU rural areas.

The case of the Baltic States in the period of 2000–2020 clearly indicates that there is a following link between employment rate and rural development (characterized by the GDP per capita): when the level of rural employment increases do does the economic growth in rural areas. This has a wider impact for a public debate on the issue of creating jobs in rural areas which then positively impact the level of rural development.

The results of the study are important for academia, policymakers and practitioners. Not only does it show the theoretical considerations on selected aspects regarding to jobs and growth in rural areas but also it focuses on its practical usage in the context of policy making issues which are then crucial for practitioners who live and/or work in rural areas.
